# Supplementary material for: Causal Relationship between Aging and Anorexia Nervosa: A White-Matter-Microstructure-Mediated Mendelian Randomization Analysis
Source: Biomedicines. 2024 Aug 16;12(8):1874. doi: 10.3390/biomedicines12081874 (PMC11351342; doi:10.3390/biomedicines12081874)
Supplement: Supplementary file 1 [file biomedicines-12-01874-s001.zip › Supplementary Figure/Figure S4/Singe SNP plot of MR analysis for TL to ubm-b-1527.pdf]

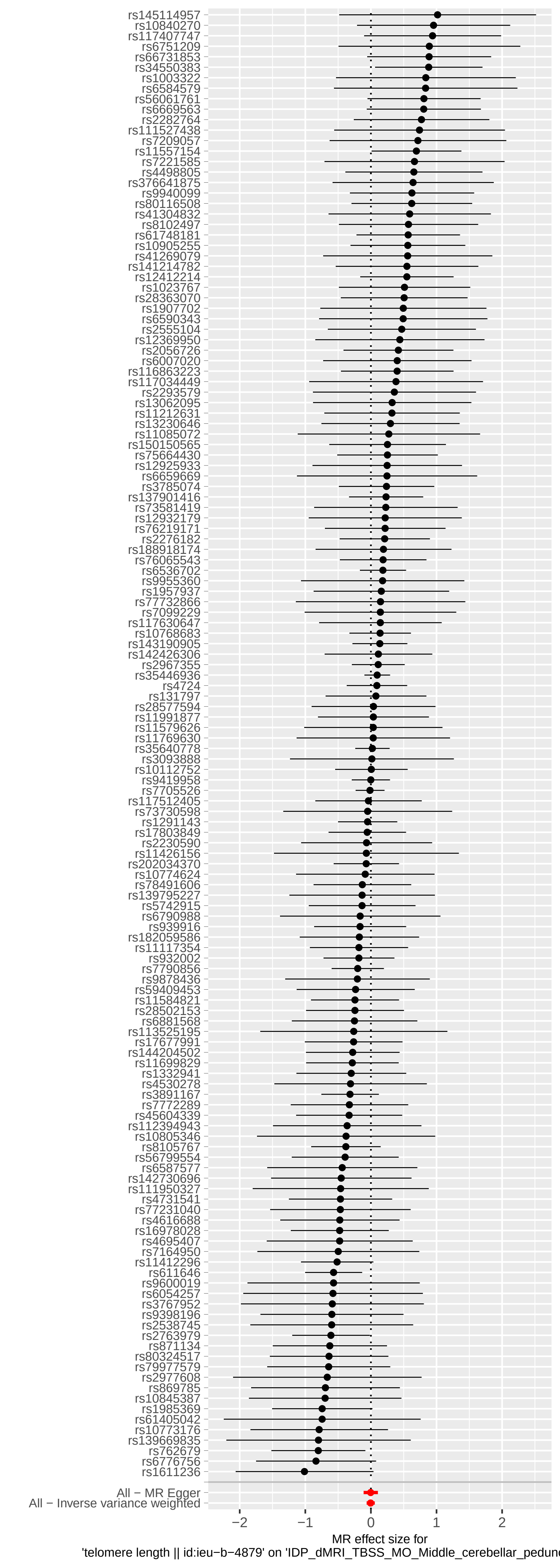

rs145114957
rs10840270
rs117407747
rs6751209
rs66731853
rs34550383
rs1003322
rs6584579
rs56061761
rs6669563
rs2282764
rs111527438
rs7209057
rs11557154
rs7221585
rs4498805
rs376641875
rs9940099
rs80116508
rs41304832
rs8102497
rs61748181
rs10905255
rs41269079
rs141214782
rs12412214
rs1023767
rs28363070
rs1907702
rs6590343
rs2555104
rs12369950
rs2056726
rs6007020
rs116863223
rs117034449
rs2293579
rs13062095
rs11212631
rs13230646
rs11085072
rs150150565
rs75664430
rs12925933
rs6659669
rs3785074
rs137901416
rs73581419
rs12932179
rs76219171
rs2276182
rs188918174
rs76065543
rs6536702
rs9955360
rs1957937
rs77732866
rs7099229
rs117630647
rs10768683
rs143190905
rs142426306
rs2967355
rs35446936
rs4724
rs131797
rs28577594
rs11991877
rs11579626
rs11769630
rs35640778
rs3093888
rs10112752
rs9419958
rs7705526
rs117512405
rs73730598
rs1291143
rs17803849
rs2230590
rs11426156
rs202034370
rs10774624
rs78491606
rs139795227
rs5742915
rs6790988
rs939916
rs182059586
rs11117354
rs932002
rs7790856
rs9878436
rs59409453
rs11584821
rs28502153
rs6881568
rs113525195
rs17677991
rs144204502
rs11699829
rs1332941
rs4530278
rs3891167
rs7772289
rs45604339
rs112394943
rs10805346
rs8105767
rs56799554
rs6587577
rs142730696
rs111950327
rs4731541
rs77231040
rs4616688
rs16978028
rs4695407
rs7164950
rs11412296
rs611646
rs9600019
rs6054257
rs3767952
rs9398196
rs2538745
rs2763979
rs871134
rs80324517
rs79977579
rs2977608
rs869785
rs10845387
rs1985369
rs61405042
rs10773176
rs139669835
rs762679
rs6776756
rs1611236
All − MR Egger
All − Inverse variance weighted
−2
−1
0
1
2
MR effect size for
'telomere length || id:ieu−b−4879' on 'IDP_dMRI_TBSS_MO_Middle_cerebellar_pedunc
